# The ultrasound risk stratification systems for thyroid nodule have been evaluated against papillary carcinoma. A meta-analysis

**DOI:** 10.1007/s11154-020-09592-3

**Published:** 2020-09-21

**Authors:** Pierpaolo Trimboli, Marco Castellana, Arnoldo Piccardo, Francesco Romanelli, Giorgio Grani, Luca Giovanella, Cosimo Durante

**Affiliations:** 1 Clinic for Nuclear Medicine and Competence Center for Thyroid Diseases, Imaging Institute of Southern Switzerland, Ente Ospedaliero Cantonale, Via Ospedale 12, 6500 Bellinzona, Svizzera Switzerland; 2 Biomedical Sciences, Università della Svizzera Italiana (USI), Lugano, Switzerland; 3 National Institute of Gastroenterology “Saverio de Bellis”, Research Hospital, Castellana Grotte, Bari, Italy; 4 Department of Nuclear Medicine, Galliera Hospital, Genoa, Italy; 5 Department of Experimental Medicine, “Sapienza” University of Rome, Rome, Italy; 6 Department of Translational and Precision Medicine, Sapienza University of Rome, Rome, Italy; 7 Clinic for Nuclear Medicine, University Hospital and University of Zurich, Zurich, Switzerland

**Keywords:** Thyroid, Ultrasound, TIRADS, Papillary, Follicular, Medullary

## Abstract

Thyroid imaging reporting and data systems (TIRADS) are used to stratify the malignancy risk of thyroid nodule by ultrasound (US) examination. We conducted a meta-analysis to evaluate the pooled cancer prevalence and the relative prevalence of papillary, medullary, follicular thyroid cancer (PTC, MTC, and FTC) and other malignancies among nodules included in studies evaluating their performance. Four databases were searched until February 2020. Original articles with at least 1000 nodules, evaluating the performance of at least one TIRADS among AACE/ACE/AME, ACR-TIRADS, ATA, EU-TIRADS, or K-TIRADS, and reporting data on the histological diagnosis of malignant lesions were included. The number of malignant nodules, PTC, FTC, MTC and other malignancies in each study was extracted. For statistical pooling of data, a random-effects model was used. Nine studies were included, evaluating 19,494 thyroid nodules. The overall prevalence of malignancy was 34% (95%CI 21 to 49). Among 6162 histologically proven malignancies, the prevalence of PTC, FTC, MTC and other malignancies was 95%, 2%, 1%, and 1%, respectively. A high heterogeneity was found for all the outcomes. A limited number of studies generally conducted using a retrospective design was found, with possible selection bias. Acknowledging this limitation, TIRADSs should be regarded as accurate tools to diagnose PTC only. Proposed patterns and/or cut-offs should be revised and other strategies considered to improve their performance in the assessment of FTC, MTC and other malignancies.

**Electronic supplementary material:**

The online version of this article (10.1007/s11154-020-09592-3) contains supplementary material, which is available to authorized users.

## Introduction

Ultrasound (US) is recognized as the most valuable imaging modality for the assessment of malignancy risk of thyroid nodules. As largely proven during the last two decades, specific nodule’s US characteristics, such as hypoechogenicity, taller-than-wide shape, irregular margins, microcalcifications, and extrathyroidal extension, should be considered as suspicious and recommend fine-needle aspiration (FNA) [1]. However, their use as single parameters suffer from low/suboptimal sensitivity and moderate/high inter- and intra-observer variability in both recognition and reporting [2]. Therefore, US risk stratification systems for thyroid nodule (RSSs, often referred to as Thyroid Imaging Reporting And Data Systems “TIRADSs”) [3–14] have been developed with the aim of: 1) establishing a standard lexicon of nodule description; 2) defining the suspicious characteristics; 3) putting the nodule into a risk category; and 4) identifying those nodules in which FNA is indicated also considering the size. Since the introduction of these RSSs in clinical practice, several studies aimed to compare their performance. However, one meta-analysis on this topic showed that, regardless of the high emphasis on RSSs, only sparse data were available in the literature, limiting the number of head-to-head comparisons that could be performed [15]. In addition, some methodological limitations are present in these studies. Ideally, we should validate these systems in a cohort as truthful as possible. Such a study should: 1) contain nodules randomized to undergo FNA or not; 2) include a histologic diagnosis to confirm or not the cytological assessment (it is recognized that FNA suffers from false positives and false negatives [9, 12]); and 3) be conducted by differently experienced US operators (radiologists and endocrinologists). Unfortunately, the published data on this topic are almost all retrospective, neither from this study design nor from this patients’ setting, and the indication to surgery was frequently based on FNA. In particular, the latter issue represents a major selection bias because FNA accurately diagnoses most papillary carcinomas (PTC) while follicular carcinoma (FTC) is invariably put in the indeterminate FNA category [16, 17] and medullary carcinoma (MTC) is misdiagnosed on FNA in up to 50% of cases [18]. In addition, the cancer prevalence in these studies varied largely and this influenced the results, since it is well known that the performance of a diagnostic test depends on the event/disease frequency [15, 19].

The present study was conceived to verify whether the performance of RSSs has been adequately investigated in all thyroid malignancies. Here we systematically searched studies classifying thyroid nodules according to five commonly used US RSSs and reporting the histological diagnosis of malignant lesions. Also, we performed a meta-analysis of available data to evaluate: 1) the pooled cancer prevalence; and 2) the relative prevalence of PTC, FTC, MTC and other malignancies.

## Methods

The systematic review was performed in accordance with the Meta-analysis Of Observational Studies in Epidemiology (MOOSE) (Supplementary Table 1) [20].

### Search strategy

A six-step search strategy was planned. Firstly, sentinel studies were searched in PubMed. Secondly, keywords and MeSH terms were identified in PubMed. Thirdly, in order to test the strategy, the terms “AACE/ACE/AME”, “ACR TIRADS”, “EU-TIRADS”, “K-TIRADS” and “ATA” were searched in PubMed. Fourthly, PubMed, CENTRAL, Scopus and Web of Science were searched. Fifthly, studies meeting all the following criteria were included: 1) at least 1000 nodules should be assessed; 2) nodules should be classified according to at least one US RSS among American Association of Clinical Endocrinologist/American College of Endocrinology/Associazione Medici Endocrinologi (AACE/ACE/AME) [10], American College of Radiology (ACR-TIRADS) [11], 2015 American Thyroid Association (ATA) [12], European Thyroid Association (EU-TIRADS) [13], and Korean Society of Thyroid Radiology and Korean Society of Radiology (K-TIRADS) [14]; 3) data on the performance of at least one of the above US RSS should be reported (e.g. the prevalence of malignancy in each US RSS class or indication to FNA according to US RSS); 4) the diagnosis of malignant nodules had not to be based on cytology only; 5) data on the overall prevalence of malignancy and the relative prevalence of PTC, FTC, MTC and other malignancies among all malignancies should be reported. Studies were excluded if focusing on pediatric patients or on specific subgroups of thyroid nodules (e.g. indeterminate, only solid or predominantly solid). Finally, references of included studies were screened for additional papers. The last search was performed on February 1st, 2020. Articles in all languages were accepted and with no restriction to the year they were published. Two investigators (MC, PT) independently and in duplicate searched papers, screened titles and abstracts of the retrieved articles, reviewed the full-texts and selected articles for their inclusion.

### Data extraction

The following information was extracted independently and in duplicate by two investigators (MC, PT) in a piloted form: 1) general information on the study (author, year of publication, country, study type, number of patients, number of nodules, selection criteria of included nodules); 2) reference standard for the diagnosis of malignancy; 3) number of malignant nodules; 4) number of PTC, FTC, MTC and other malignancies. The main paper and supplementary data were searched; if data was missing, authors were contacted via email. Data were cross-checked and any discrepancy was discussed.

### Study quality assessment

The risk of bias of included studies was assessed independently by two reviewers (MC, PT). The National Heart, Lung, and Blood Institute Quality Assessment Tool was used, and the following aspects evaluated: study question; eligibility criteria; sample size calculation; description and delivering of intervention; definition of outcome measures; duration of follow-up; blinding; loss to follow-up; statistical methods. Each domain was assigned absence, unclear or possible risk of bias or as not applicable [21].

### Data analysis

The characteristics of included studies were summarized. Then, separate analyses were performed according to the following steps. First, a proportion meta-analysis was carried to obtain the pooled prevalence of malignancy among all included nodules. A sub-group analysis was performed for studies including histologic series only or both histologic and cytologic series. Second, a proportion meta-analysis was carried to obtain the pooled prevalence of PTC, FTC, MTC and other malignancies among malignancies diagnosed at histology. Heterogeneity between studies was assessed by using I^2^, with 50% or higher values regarded as high heterogeneity. The Egger’s test was carried out to evaluate the possible presence of significant publication bias; the trim-and-fill method was used for estimating its effect. For statistical pooling of data, a random-effects model was used. All analyses were performed on a per lesion basis and carried out using StatsDirect statistical software (StatsDirect Ltd.; Altrincham, UK) and Prometa3.0 (Internovi). A *p* < 0.05 was regarded as significant.

## Results

**Fig. 1:**
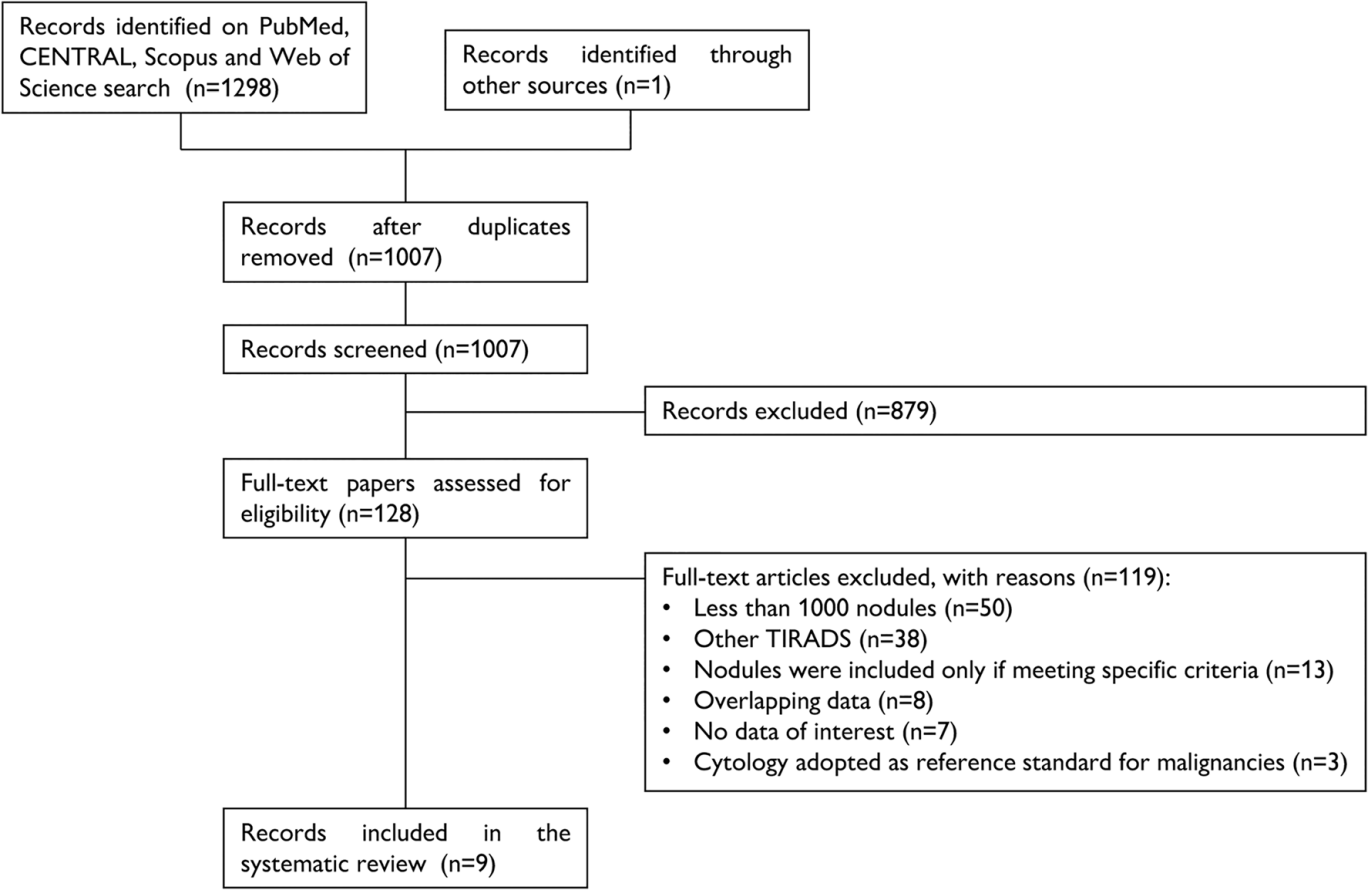
Flow chart of the systematic review

A total of 1298 papers were found, of which 193 on PubMed, 56 on CENTRAL, 155 on Scopus, and 894 on Web of Science. One additional paper was retrieved from a personal database [22]. After removal of 292 duplicates, 1007 articles were analyzed for title and abstract; 879 records were excluded (guidelines, review, meta-analysis, inclusion of specific subgroups of nodules, pediatric patients, not within the field of the review). The remaining 128 papers were retrieved in full-text and 9 studies were finally included in the systematic review (Fig. 1) [22–30]. No additional study was retrieved from references of included studies.

### Study quality assessment

The risk of bias of the included studies is shown in Supplementary Table 2. Statement of the study question, description of the study population, participation rate, assessment of the exposures and outcome bias were adequate in all. Ultrasound was performed before cytology or surgery and images retrospectively reviewed but the timeframe between the two assessments was considered as adequate as cancer a chronic disease. Reviewers were generally blinded to the final diagnosis.

### Qualitative analysis (systematic review)

The characteristics of the included articles are summarized in Table 1. The studies were published between 2017 and 2020 and had sample sizes ranging from 1001 to 4696 thyroid nodules. All studies were retrospective cohort and assessed the performance of at least one TIRADS among AACE/ACE/AME, ACR-TIRADS, ATA, EU-TIRADS, K-TIRADS. Five studies were carried out in China, two in the United States of America, one in Korea, and one multicenter study in France, Switzerland and the United Kingdom. Participants were adult outpatients with US images available who had undergone either thyroid nodule FNA or surgery in six studies or surgery only in three studies [24, 26, 27]. Thyroid nodules diagnosed as non-diagnostic or indeterminate on FNA were excluded, unless a final diagnosis was met on pathology. Overall, 19,494 thyroid nodules were included in the present review, among which 6391 were malignant. Among the 6162 malignant nodules diagnosed at histology, the number of PTC, FTC, MTC and other malignancies was 5963, 97, 54 and 48, respectively.

**Table 1:** Characteristics of included studies

First Author, year [ref]	Country	Study design	Thyroid nodules (n)	Selection criteria of included nodules	Reference standard for malignancy
Yoon, 2017 [22]	Korea	RCS	4696	10–19 mm, benign cytology, malignant cytology or surgery	Histology or cytology
Middleton, 2018 [23]	United States of America	RCS	3422	Benign cytology, malignant cytology or surgery	Histology or cytology
Gao, 2019 [24]	China (Beijing)	RCS	2544	Surgery	Histology
Ruan, 2019 [25]	China (Guangzhou)	RCS	1001	Benign cytology, malignant cytology or surgery	Histology or cytology
Shen, 2019 [26]	China (Shanghai)	RCS	1612	>5 mm, surgery	Histology
Trimboli, 2019 [27]	France, Switzerland, United Kingdom	RCS	1058	≥5 mm, surgery	Histology
Wildman-Tobriner, 2019 [28]	United States of America	RCS	1425	Benign cytology, malignant cytology or surgery	Histology
Xu, 2019 [29]	China (Nanjing)	RCS	2465	Benign cytology or surgery	Histology
Zhang, 2020 [30]	China (Shanghai)	RCS	1271	≥5 mm, benign cytology, malignant cytology or surgery	Histology or cytology

Legend – *RCS* retrospective cohort study

### Quantitative analysis (meta-analysis)

The overall prevalence of malignancy in all articles included in the meta-analysis was 34% (95%CI 21 to 49). When a subgroup analysis according to the reference standard for malignancy was performed, no difference was found between studies using histology only or cytology and histology (37%; 95%CI 18 to 57 versus 31%; 95%CI 14 to 51, respectively; *p* = 0.64).

**Fig. 2:**
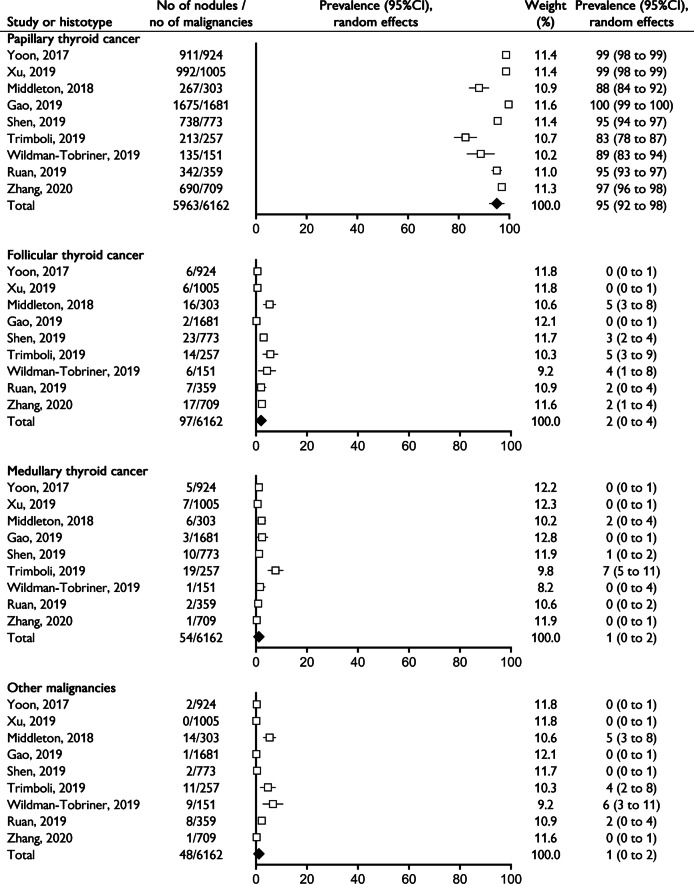
Forest plot of the four meta-analyses of the relative prevalence of papillary, follicular, medullary thyroid cancer and other malignancies among histologically proven malignancies

Among the 6162 histologically proven malignancies, four separate meta-analyses on the prevalence of PTC, FTC, MTC and other malignancies were performed and it was found a rate of 95%, 2%, 1%, and 1%, respectively (Fig. 2). A high heterogeneity and evidence of publication bias were found for all the outcomes, with the exception of the overall prevalence of malignancy; the trim-and-fill method did not change the estimates (Supplemental Table 3).

## Discussion

Thyroid US examination represents the gold standard for the management of thyroid nodules, their risk stratification, and their indication for FNA. With the present article we raised the question of whether the RSSs have been evaluated for all types of thyroid malignancies. Accordingly, we browsed the published literature to find the largest number of original papers, with a minimum simple of one thousand nodules, which aimed to verify the accuracy of RSSs and included histologically diagnosed malignant lesions. Two main questions were addressed in our study: 1) what is the cancer rate reported in these studies? and 2) what is the relative prevalence of the histologic types of thyroid malignancy? With the introduction of the RSSs in clinical practice all thyroidologists started to select thyroid nodules for FNA or clinical follow-up according to the criteria described in these consensus or guidelines [3–14]. The latter should represent a significant advancement of thyroid US culture towards a homogeneous worldwide approach to thyroid nodule [31]. However, before considering the RSSs as the gold standard to manage our patients we should have more solid proofs and be aware of what we can reasonably expect from these systems. In fact, one thyroidologist using any RSSs might expect that these systems have been designed to identify all types of thyroid malignancies. The results of our study challenge this expectation.

According to our search strategy we found nine articles including a total of 19,494 thyroid nodules of which 6391 were malignant. The pooled cancer prevalence reported in these articles was 34%, with heterogeneity. Moreover, among all malignant nodules, PTC represented the 95%. Both findings are of high clinical relevance. First, all RSSs were conceived for selecting thyroid nodules for FNA. Then, when comparing their performance, only summary operating measures assumed to be independent of the disease prevalence should be used (e.g. diagnostic odds ratio) [15]. On the other hand, biased result can be obtained if a comparison is based only on other parameters (e.g. sensitivity, specificity) [32]. Second, from a clinical point of view, this histologic prevalence deserves more thorough discussion. What is particularly striking is that the percentage of FTC and MTC seems much lower than expected in such selected populations [33]. This finding can be due to the challenges faced by clinicians when making a diagnosis of FTC or MTC. FTC has often an unsuspicious echo-structural presentation and cannot reliably be diagnosed on cytology, as stated [17]. Therefore, cytologically indeterminate thyroid nodules without suspicious sonographic patterns warrant particularly careful follow-up strategies. In addition, FTC rate is heavily influenced by the epidemiological curves, which are consistently showing a decrease in the frequency of new cases over the last three decades [34, 35]. Similarly, MTC has an heterogenous US presentation and is difficult to detect on FNA [17, 36]. The routinely use of serum calcitonin, the most sensitive tool for MTC diagnosis, can possibly improve diagnosis, but it is still a debated matter [4, 7, 10, 12]. Finally, the rate of the other thyroid malignancies (i.e., lymphoma, metastases from other organs) is expected low and these lesions may have heterogeneous US presentation too [37]. It has also to be taken into account that PTC is the most frequently diagnosed thyroid cancer, it can be easily detected in the clinical practice due to its typical US features and the high performance of the cytological assessment. Therefore, when reviewing a histological series of thyroid nodules, a large number of PTC is widely expected. All these clinical issues may have affected the relative rates of the different types of thyroid malignancies included in the studies evaluating the accuracy of RSSs.

Beyond all the above considerations, it is indisputable based on our data that RSSs’ performance has been tested almost exclusively in PTC patients, thus supporting the view that the clinical validity of these systems cannot be unconditionally extended to other forms of malignancy [31]. Therefore, while a sonographic-centered diagnostic work-up can effectively identify PTCs, RSSs cannot be advocated to reliable diagnose those cancers burdened by the greatest risk of mortality, i.e., FTCs [16] and MTCs [38]. Patients diagnosed with a large FTC have a higher risk of developing distant macro-metastases, for which radioactive iodine therapy may be ineffective [39]. MTCs are expected to spread-out early to loco-regional lymph-nodes and to distant sites, even if diagnosed when small in size [40]. All cases falling in these clinical scenarios are invariably not curable, require lifelong treatments often affecting the quality of life, and have a lower life expectancy [41]. An important diagnostic effort should be fielded to allow clinicians not to miss these diagnoses. This implies an effort to develop US RSSs able to intercept FTC and MTC cases. On the other hand, a great effort is underway in validating molecular tests to improve cytological diagnosis [42–45]. In the meantime, international guidelines have sped up this process by promoting the potential of molecular tests in clinical practice [12, 41].

This review has several limitations. The first limitation relates to the design of included studies: a retrospective review of nodules that have been submitted to FNA or surgery was performed in most of them, and this introduced a significant selection bias. We selected only those studies in which at least 1000 nodules were included, and this is a second limitation. However, the prevalence of FTC, MTC and other cancers is expected to be low compared to PTC, then only studies with an adequate sample size could be deemed sufficiently powered to reliably determine their frequency. Lastly, despite being classified as PTC, specific subtypes have been associated with a worse prognosis [12]. Further studies are needed to assess the representativeness of these subtypes in studies assessing the performance of US RSSs.

In conclusion, almost all histologically proven cancers found in the studies evaluating the accuracy of RSSs are PTCs. On one hand, this suggests that US classifications are an accurate tool to diagnose PTC. Their reliability in detecting FTC, MTC and other malignancies should still be improved, by either modifying patterns and cut-offs for FNA or integrating US with other technologies. From another perspective, our results raise the question of whether during our clinical practice we are on the hunt of PTCs while we are neglecting the most aggressive thyroid cancers. We advise for further studies investigating the latter issue.

## Electronic supplementary material

ESM 1 (DOCX 27 kb)

## Data Availability

The datasets generated during and/or analyzed during the current study are not publicly available but are available from the corresponding author on reasonable request.
